# Potential Pathophysiological Pathways in the Complex Relationships between OSA and Cancer

**DOI:** 10.3390/cancers15041061

**Published:** 2023-02-07

**Authors:** Manuel Sánchez-de-la-Torre, Carolina Cubillos, Olivia J. Veatch, Francisco Garcia-Rio, David Gozal, Miguel Angel Martinez-Garcia

**Affiliations:** 1 Group of Precision Medicine in Chronic Diseases, Respiratory Department, University Hospital Arnau de Vilanova and Santa María, Department of Nursing and Physiotherapy, Faculty of Nursing and Physiotherapy, IRBLleida, University of Lleida, 25003 Lleida, Spain; 2 Centro de Investigación Biomédica en Red de Enfermedades Respiratorias (CIBERES), Instituto de Salud Carlos III (ISCIII), 28029 Madrid, Spain; 3 Group of Respiratory Diseases, Respiratory Department, Hospital Universitario La Paz-IdiPAZ, 28029 Madrid, Spain; 4 Department of Psychiatry and Behavioral Sciences, University of Kansas Medical Center, Kansas City, KS 66103, USA; 5 Department of Child Health and Child Health Research Institute, University of Missouri School of Medicine, Columbia, MO 65212, USA; 6 Department of Medical Pharmacology and Physiology, University of Missouri School of Medicine, Columbia, MO 65212, USA; 7 Respiratory Department, University and Polytechnic La Fe Hospital, 46026 Valencia, Spain; 8 Pneumology Department, University and Polytechnic La Fe Hospital, 46012 Valencia, Spain

**Keywords:** obstructive sleep apnea, tumor, cancer, malignancy, intermittent hypoxia, exosomes, microbiome, biomarker, immune system

## Abstract

**Simple Summary:**

Previous clinical studies have suggested a relationship between obstructive sleep apnea and some types of cancer. This relationship is very heterogeneous, however. This heterogeneity probably depends on the complex pathophysiological pathways activated. It seems that the hypoxia inducible transcription factor, a molecule expressed in situations of hypoxemia such as obstructive sleep apnea, is a key factor. In recent years, however, some new pathophysiological pathways have been shown to be involved, including a large number of biomarkers, pathways related to immune cell dysfunction, exosomes, genetics, and even microbiome alterations. Furthermore, there is an array of tumor cell lines, each with a different capacity to respond to intermittent hypoxia. Both clinical and murine model studies suggest that tumors, such as melanoma and some histological types of breast and lung cancer, could respond in different ways from cancer cell lines in the prostate or the liver.

**Abstract:**

Several epidemiological and clinical studies have suggested a relationship between obstructive sleep apnea (OSA) and a higher incidence or severity of cancer. This relationship appears to be dependent on a myriad of factors. These include non-modifiable factors, such as age and gender; and modifiable or preventable factors, such as specific comorbidities (especially obesity), the use of particular treatments, and, above all, the histological type or location of the cancer. Heterogeneity in the relationship between OSA and cancer is also related to the influences of intermittent hypoxemia (a hallmark feature of OSA), among others, on metabolism and the microenvironment of different types of tumoral cells. The hypoxia inducible transcription factor (HIF-1α), a molecule activated and expressed in situations of hypoxemia, seems to be key to enabling a variety of pathophysiological mechanisms that are becoming increasingly better recognized. These mechanisms appear to be operationally involved via alterations in different cellular functions (mainly involving the immune system) and molecular functions, and by inducing modifications in the microbiome. This, in turn, may individually or collectively increase the risk of cancer, which is then, further modulated by the genetic susceptibility of the individual. Here, we provide an updated and brief review of the different pathophysiological pathways that have been identified and could explain the relationship between OSA and cancer. We also identify future challenges that need to be overcome in this intriguing field of research.

## 1. Introduction

Despite the phenotypic heterogeneity of obstructive sleep apnea (OSA) [1,2], two pathophysiological consequences that especially determine its systemic impact on the individual—from the cardiovascular [3,4,5,6,7,8,9,10,11,12,13,14,15,16], metabolic [17,18], and neurocognitive points of view [19,20]—are intermittent hypoxemia (IH) and sleep fragmentation (SF) [21]. In recent years, it has been observed that both IH and SF can trigger other pathophysiological mechanisms that may explain the association between different sleep-related disorders (especially OSA) and a higher prevalence, incidence, and aggressiveness of some types of cancer [21,22]. One of the first pathophysiological pathways uncovered included an increase in the expression and circulating levels of vascular endothelial growth factor (VEGF), which was induced by the up-regulation of hypoxia inducible factor (HIF-1α) transcriptional activity as a consequence of hypoxemia. This increase in VEGF was associated with greater neovascularization of the tumor and a higher probability of the metastatic spread in some cancers [23]. Since then, additional mechanisms have been uncovered that may help explain the complex relationship between OSA and cancer. These mechanisms involve genetic, molecular, cellular, and even microbiological determinants [24] (Table 1). The substantial diversity of these pathophysiological pathways and their involvement in metabolism and other intra-cellular processes of different malignant cell types likely explain the heterogeneity in the relationship between OSA and different histological types and locations of cancer [25]. In this manuscript, we offer a brief review of the different pathophysiological mechanisms that are currently being studied and may explain the possible relationship between OSA and cancer.

## 2. Quantitative or Qualitative Cell Dysfunction

OSA and its cognate manifestation of IH have been shown to induce several alterations in the immune system response, likely through the generation and propagation of reactive oxygen species (ROS) and consequent oxidative stress. In the context of exploring mechanisms underlying various frequent OSA-related morbidities, several authors have proposed that IH serves as a parallel reporter of OSA-related end-organ dysfunction and that such perturbations include a disturbance of the immunosurveillance system due to both immune cell dysfunction and other alterations in the immune microenvironment [26,27,28,29,30,31,32,33,34,35,36]. All of these consequences may favor the development and progression of cancer.

Thus far, the exact nature of the pathophysiological processes underlying the modulation of immune cell function in OSA patients has not been fully elucidated; however, there is solid evidence that increased inflammatory conditions are present and detectable in OSA patients, animal models, and in vitro assays. Indeed, several lines of evidence suggest that inflammatory conditions may serve as a substrate that initiates most OSA-induced comorbidities. Along these lines, chronic inflammation has been conclusively linked to a multistep sequence of processes involved in the progressive transformation of normal cells into malignant cancer cells [37]. Indeed, there is solid evidence to support the relationship between hypoxic conditions and inflammatory response in various immune subsets [38,39]. In particular, the HIF-1α promotes inflammatory cytokines via the master transcription factor in the inflammatory response, NF-κB [40,41,42]. Along these lines, the first large study in OSA subjects showed a significant relationship between the NF-κB-dependent genes TNFα and IL-8 and hypoxemic clinical indicators [30]. A later study using OSA monocytes showed that the NF-κB protein increased the nuclear translocation of phosphorylation, demonstrating NF-κB activation [35]. For example, pro-inflammatory responses are dependent on TLR4/NF-κB under IH conditions in both bone marrow-derived macrophages cells (BMDM) from mice and monocytes from OSA patients [43,44,45,46,47]. Moreover, NF-κB activation and transcriptional activity enhance the expression of pro-inflammatory cytokine IL-6 [48,49,50]. IH has also been related to high levels of cytokine IL-1β in murine cells [43,51,52], and the subsequent loss of NLRP3 under such exposure reduced IL-1β expression in murine BMDM [53]. Furthermore, it has been demonstrated in OSA patients that IH contributes to the activation of the NLRP3 inflammasome in parallel with NF-κB activation and a resulting increase in a large number of inflammatory cytokine levels [40]. Collectively, these data suggest the upregulation of inflammatory pathways by OSA and IH ultimately results in the release of clusters of inflammatory cytokines that exert a multiplicity of functions downstream.

Moreover, macrophage populations are highly susceptible to changing their phenotype in response to the microenvironment. Two major phenotypes that have been established are the classically activated phenotype M1 and alternatively activated phenotype M2. The M1 macrophage population is described as a pro-inflammatory phenotype, whereas M2 is characterized by other functions, including anti-inflammatory signaling [53,54]. Notably, several studies conducted in murine models of OSA have found that under IH conditions, the number of pro-inflammatory M1 macrophages is increased, while the population of anti-inflammatory M2 macrophages is diminished [55,56,57,58]. Several approaches have been attempted to elucidate the pathophysiological pathways driving inflammation and cancer, while also promoting OSA comorbidities as a function of increases in the M1 macrophage phenotype. On the one hand, low-grade systemic inflammation related to obesity promotes cancer progression by remodeling the immune cell landscape [54,55]. In this particular setting, IH leads to altered adipose and vascular tissues [57,59,60,61,62,63], and M1 macrophage polarization promotes adipogenic differentiation in lean mice [64]. This pathophysiological mechanism, including subcutaneous adipose tissue remodeling and inflammation, increases visceral and liver fat deposition. This results in metabolic dysfunction under IH, and when sleep fragmentation is implemented in animal models to mimic the recurrent arousals that occur in OSA patients [63,65,66]. On the other hand, growing evidence has shown that inflammation impacts the tumor microenvironment and the plasticity of tumor and stromal cells [67]. Moreover, IH exposures have been observed to promote the emergence of stable genotypic and phenotypic properties that can readily promote tumor progression [68], and affect the interplay between tumor cells and stromal cells by the COX-2/PGE2 signaling pathway [69]. Intriguingly, increased M2 macrophage subsets have been repeatedly identified in tumor sites of mice exposed to IH, suggesting that within the tumor setting, the effect of IH may differ from those on circulating macrophages in non-malignant tissues [70,71].

It should be noted that major antigen-presenting cells (e.g., dendritic cells) play a crucial role in the early detection of cells that have undergone neoplastic transformation and their subsequent eradication via T-cell lymphocytes. However, their presence is relatively low in the blood circulation, making it difficult to study dendritic cells in OSA patients [72]. In a study by Galati and colleagues, a reduction in dendritic cell frequency related to inflammatory cytokine (IL-6) was detected in OSA patients, when compared with healthy donors [73]. Lastly, other innate immune subsets that might be potentially involved in the relationship between OSA and cancer are NK cells and γδ T cells. While Dyugovskaya and colleagues demonstrated an amplification of inflammatory phenotypes in OSA patients, characterized by an increase in the avidity and cytotoxicity of γδ and CD8 T cells against an endothelial cell line [74,75], γδ T-cell populations from OSA patients showed reduced expression of perforin (related to cytotoxicity), which may facilitate a higher cancer incidence [76]. Cytotoxic activity in NK cells is also reduced in OSA patients, as shown by a reduced CD3^−^CD56^+^CD16^+^ frequency and decreased cytotoxic activity [77]. Moreover, the iNKT subset is less abundant in OSA subjects and correlated with several disease severity measures (e.g., apnea/hypopnea index, oxygen desaturation index, and overall nocturnal hypoxemia) [78]. Collectively, a reduction in indicators of cytotoxic effectors in OSA and IH may point to a pathophysiological pathway connecting innate immune dysfunction caused by OSA with a risk of cancer.

The adaptive immune response is classically represented by T cells responsible for tumor immunosurveillance and infection control. In this respect, a pathophysiological link between cancer and OSA incidence has been reported, involving impaired T-cell function. In an OSA animal model, low levels of granzyme B in T-cell lymphocytes emerged, along with decreased cytotoxicity of tumor-infiltrating CD8 T lymphocytes (CTLs), when compared with the animal control group [79]. The authors postulated that the impaired function of effector CTLs enables cancer cell immune escape. In this regard, data from OSA patients and in vitro models suggested that IH enhances the expression of transcription factors (TFs) related to epithelial–mesenchymal transition (EMT), such as TWIST, SNAIL, and SLUG, as well as promoting the de-differentiation of cancer cells to cancer stem cells (CSC) through the increased expression of embryonic stem cell transcriptional regulators, such as OCT4, SOX2, and NANOG. This was proposed to further invoke an avenue enhancing the pathophysiological link between tumor progression and IH [36]. This effect of IH on the acquisition of CSC and EMT features is mediated by the modulation of transforming growth factor β (TGF-β) signaling through paraspeckle component 1 (PSPC1) [11]. Indeed, in cutaneous melanoma patients, OSA severity is associated with higher PSPC1 serum levels, which—jointly with IH—would enhance the self-reprogramming capabilities of the EMT and CSC feature acquisition of melanoma cells, promoting their intrinsic aggressive properties [80].

So far, studies focusing on circulatory T-cell dynamics in OSA patients have demonstrated an increase in the immune checkpoint programmed cell death receptor1 (PD-1) in CD4 and CD8 T cells [33]. These authors speculated that hypoxemia induced by IH may promote high levels of the receptor and facilitate impairments in T-cell function [33]. This study also revealed the programmed cell death ligand 1 (PD-L1) upregulation in monocytes from patients with OSA and healthy monocytes subjected to IH exposures [33]. Other studies have corroborated the findings that OSA patients exhibited high levels of PD-1/PD-L1 [32,34,81,82,83]. Following these key PD-1/PD-L1 data, studies in patients with cutaneous melanoma and OSA observed that plasma-soluble PD-L1 (sPD-L1) correlated with melanoma aggressiveness based on the Breslow index, in both advanced primary tumor stages and patients with locoregional disease. Strikingly, the addition of sPD-L1 to the classic risk factors for predicting sentinel lymph node metastasis led to net improvements in the classification of 27.3% of cases [84]. This cumulative evidence provides insight into how reduced or ineffective T-cell immunosurveillance caused by IH promotes an increase in PD-1/PD-L1 crosstalk, ultimately facilitating tumor escape and eventual tumor progression; and thus, highlighting a potential pathophysiological pathway between elevated cancer incidence for specific tumors in OSA patients.

## 3. Role of Specific Biological Biomarkers

The definition of, and potential for biomarkers to detect disease states has been extensively discussed. Briefly, a biomarker can be described as “a defined characteristic that is measured as an indicator of normal biological processes, pathogenic processes or responses to an exposure or intervention” [85]. The role of a biomarker not only has functionality in the clinical management of disease, but also informs the pathogenic pathways of a disease, reveals alterations in homeostatic regulation, and identifies mechanisms associated with a biological process underlying the disease. The potential clinical utility of biomarkers in cancer has been extensively explored. In recent years, a large number of studies have emerged exploring the role of IH in the promotion and function of specific biomarkers related to cancer progression. It has been largely reported that hypoxia plays an important role in the association of OSA and cancer, as it is a key pathophysiological mechanism that promotes the development of many inflammatory and tumor diseases. Interestingly, the potential role of hypoxia is not homogeneous, and significant differences in basic tolerance to oxygen deficiency have been reported. The differences in the tolerance of or susceptibility to hypoxia can be objectivated through the evaluation of specific biomarkers; thus, determining predisposition to the development of hypoxia-related disorders. Heterogeneous responses to hypoxia would impact the varying developments of systemic and local inflammatory diseases. In fact, HIF may provide both pro-inflammatory and anti-inflammatory functions, and different isoforms of HIF can play different roles in the development of inflammatory and tumor diseases. Hypoxia tolerance may determine predisposition to the development of certain infectious, inflammatory, and tumoral processes, along with other well-known factors such as age, gender, and ethnicity, among others [26,70,86].

Previous studies have identified biomarkers of tumor aggressiveness at both the histological and systemic levels (Table 2). Specifically, the expression of HIF-1α has been related to an increase in the aggressiveness of cutaneous melanoma in patients with OSA. In fact, an association was observed between the levels of this biomarker in tumor cells and specific OSA variables, such as the desaturation index [87]. This association was also significant in relation to the Breslow index and sentinel node involvement [88]. The relationship between the expression levels of specific target genes regulated by HIF-1α and the aggressiveness of tumors in patients with OSA has also been explored. More specifically, research has been undertaken in the levels of VEGF (which have been widely recognized as promoting tumor growth and metastasis) as an inducer of angiogenic processes [89,90]. The available evidence does not identify any significant relationship between the levels of this biomarker and OSA variables, such as AHI, hypoxemia variables, and melanoma aggressiveness [91]. These findings have been corroborated by different studies, which have essentially pointed out the inconsistency between VEGF levels and the malignancy of the tumor [92,93]. This apparent discrepancy between the role of VEGF in cancer biology and its utility as an indicator of cancer aggressiveness needs to be clarified in future studies, so that the implications of IH associated with OSA in the expression of HIF-1α and VEGF at the tumor level can be better understood.

Among the markers associated with tumor aggressiveness, an increase in vascular cell adhesion molecule (VCAM)-1 levels has been identified in patients with concurrent OSA and melanoma, when compared with patients with melanoma alone [94]. Exploration of the levels of this biomarker could inform specific functions in tumor growth, the formation of metastatic niches, and the support of the angiogenic process.

In recent years, the application of immune checkpoint blockade therapies has revolutionized treatment for various cancers. The main objective of these treatments is to focus on the system formed by programmed death 1 (PD-1) and its ligand PD-L1 (PD-1/PD-L1), a complex that participates in several aspects of the disease, such as the growth of the tumor, aggressiveness, and survival [95,96]

HIF-1α, as previously described, has a regulatory function in PD-1/PD-L1 expression in patients with severe OSA, limiting the proliferative and cytotoxic capacity of T cells. It has recently been proposed that plasma concentrations of the soluble part of PD-L1 (sPD-L1) could be a useful biomarker for the characterization of aggressiveness and metastasis in cutaneous melanoma [33,34]. Thus, the available evidence suggests higher plasma levels of sPD-L1 in patients with OSA and melanoma. These elevated levels were also related to tumor aggressiveness and sentinel lymph node involvement [84].

Tumor growth factor beta (TGF-β) is abundantly expressed in the tumor microenvironment and plays a major pleiotropic role in cancer [97]. TGF-β is a multifactorial peptide growth factor that is synthesized by macrophages, lymphocytes, fibroblasts, platelets, epithelial, and cancer cells. This growth factor plays a prominent role in the regulation of different physiological processes—mainly proliferation, cell migration, adhesion, and tissue repair [98]. In OSA patients with cutaneous melanoma, TGF-β1 levels correlated with the mitotic index, Breslow index, and melanoma growth rate. Furthermore, plasma levels were increased in the presence of tumor ulceration or higher Clark levels (i.e., adverse prognostic clinical scores). Nevertheless, the correlation between TGF-β1 and melanoma aggressiveness was only observed in patients without obesity [99]. The available evidence indicates that the potentially deleterious effect induced by TGF-β1 is likely due to OSA-related IH. Accordingly, evidence from basic models in cell cultures in hypoxic conditions and hypoxic ischemic tissues has shown increased levels of this biomarker [100]. Interestingly, HIF-1α-mediated induction of TGF-β appears to play a key role in establishing an immunosuppressive phenotype in monocytes and natural killer cells from patients with OSA [77]. Paraspeckles are nuclear bodies located in the interchromatin space of the cell nucleus and are mainly composed of long non-coding RNA NEAT1 and three proteins, one of which is paraspeckle component 1 (PSPC1). Paraspeckle formation is dynamic and it is triggered by numerous cell stress scenarios, such as malignant transformation. It has been reported that IH mediates PSPC1 upregulation in OSA [36]. PSPC1 protein expression would be accompanied by increased levels of TGFβ, which ultimately switches from an immune surveillance role to a tumor progression function. 

The oxidant/antioxidant imbalance and increase in reactive oxygen species (ROS) in OSA have been related to a systemic increase in the concentration of pro-inflammatory substances, including tumor necrosis factor alpha (TNF-α). Increased plasmatic concentrations of this biomarker are associated with atherosclerosis, stroke, and cardiovascular disease. In addition to being involved in the systemic inflammatory response, TNF-α is involved in the control of tumor growth. The latest studies have reported consistent evidence of elevated TNF-α levels in adults, correlated with the severity of OSA [101]. Further research is needed to explore the association between increased TNF-α and tumor progression in patients with OSA and cancer.

The cyclooxygenase pathway results in the production of prostaglandins (PG) such as PGE2, through the activity of COX enzymes 1 (COX-1)-and 2 (COX-2). PGE2 plays a central role in the regulation of multiple biological processes under both normal and pathological conditions, and it has been most thoroughly investigated in cancer. COX-2 has been identified in many human cancers, pre-cancerous lesions, and metastases. It has been shown that IH induces the expression of COX-2 and results in an increased synthesis of PGE2 [71]. It has recently been hypothesized that up-regulation of the COX-2/PGE2 pathway induced by hypoxia plays a central role in the association between OSA and cancer [69].

Furthermore, the role of IH in the expression of other biomarkers related to tumor progression has also been studied. Cannabinoid receptors (CBs) are members of the G-protein-coupled receptor family and are expressed throughout the body. Specific subtypes of CBs have been reported as participants in a variety of physiological processes, and their agonists promote the proliferation of colon cancer cells and enhance an aggressive molecular feature through the activation of the AKT/GSK-3β pathway [102]. It has recently been reported that chronic IH exposure facilitates the proliferation and migration of breast cancer cells by upregulating CB1 and CB2 in vitro and in vivo, revealing a putative novel mechanism for the metastatic potential of breast cancer [103].

Anti-tumor effects of specific biomarkers have also been identified in animal models of OSA. Endostatin, the proteolytic fragment of collagen type XVIII, has been detected as a potent inhibitor of tumor angiogenesis. The therapeutic potential of endostatin in cancer has been widely investigated. Endostatin specifically inhibits endothelial proliferation and potently inhibits angiogenesis and tumor growth. As previously discussed, IH facilitates tumor growth and cancer progression in murine models. Interestingly, it has been reported that the anti-tumor effects of endostatin are more prominent when tumor-bearing mice are subjected to IH exposures mimicking OSA [104].

The available evidence supports a role for endothelin-1 and its receptors in cancer promotion under IH conditions. The endothelin axis has been shown to promote cell proliferation and migration, angiogenesis, metastasis, and chemoresistance. Interestingly, a recent study demonstrated that treatment aimed at endothelin receptor blockade prevented IH-induced tumor development in both in vitro and in vivo models [28]

In recent years, the efforts to identify biomarkers of cancer progression and their relationships with the main pathogenic consequences of OSA have made it possible to detect putative specific pathways underlying deleterious effects induced by OSA in the context of tumor growth and progression. Intervention studies carried out to date have shown a positive effect of CPAP treatment on genetic pathways related to tumorigenesis [105]. However, the impact of OSA treatment on potential biomarkers of tumor aggressiveness has not been fully elucidated, and evidence is lacking as regards any possible beneficial effect of CPAP treatment in terms of decreasing tumor aggressiveness and improving cancer outcomes in patients with OSA.

**Table 2:** Main biomarkers associated with the relationship between OSA and cancer.

Biomarker	Main Functions	Biomarker in OSA and Cancer
HIF-1α [87]	Inducer of angiogenic processes.	Inducer of angiogenic processes. Increased levels associated with aggressiveness of melanoma in patients with OSA.
VEGF [89]	Promoter of tumor growth and metastasis. Inducer of angiogenic processes.	No specific relationship between circulating levels with variables of OSA severity. Inconsistency between VEGF levels and malignancy of the tumor in OSA. Future studies must elucidate the consequences of intermittent hypoxia associated with OSA in the expression of HIF-1α and VEGF at the tumor level.
VCAM-1 [94]	Marker associated with tumor aggressiveness. Specific functions in tumor growth, formation of metastatic niches, and angiogenic process.	Increased levels of VCAM-1 in patients with OSA and melanoma.
PD-1/PD-L1 [33,84]	Complex that participates in different cancer stages. Used as immune checkpoint blockade therapies in cancer treatment.	The biomarker HIF-1α has a regulatory function in PD-1/PD-L1 expression in patients with severe OSA. Increased levels of the soluble part of PD-L1 (sPD-L1) in patients with OSA and melanoma. Potential utility as biomarker for the characterization of aggressiveness and metastasis in melanoma.
TGF-β [95]	Common expression in the tumor microenvironment. A major pleiotropic role in cancer.	In OSA patients with cutaneous melanoma, TGF-β1 levels correlated with melanoma aggressiveness, but only in patients without obesity. HIF1α-mediated induction of TGF-β appears to play a key role in establishing an immunosuppressive phenotype in monocytes and natural killer cells of patients with OSA. Basic models of hypoxia and hypoxic ischemic tissues reported increased levels of this biomarker.
PSPC1 [11]	Nuclear bodies are located in the interchromatin space of the cell nucleus. Paraspeckle formation carry on numerous cell stress scenarios such as malignant transformation.	Intermittent hypoxia mediates PSPC1 upregulation in OSA. PSPC1 protein expression would be accompanied by increased levels of TGFβ, resulting in a tumor progression function.
TNF-α [101]	A pro-inflammatory molecule with a role in systemic inflammatory response. Increased plasma concentration is associated with cardiovascular outcomes.	TNF-α plays a role in the control of tumor growth. Elevated TNF-α levels in adults are correlated with severity of OSA. Further research is needed to explore the potential association between increased TNF-a and tumor progression in patients with OSA and cancer.
COX-2/PGE2 [71]	Cyclooxygenase pathway (COX-1/COX-2) results in production of PGE2. PGE2 plays a central role in the regulation of multiple biological processes under normal and pathological conditions. COX-2 has been identified in many human cancers, precancerous lesion, and metastasis.	Intermittent hypoxia induces expression of COX-2, resulting in an increased synthesis of PGE2. A new hypothesis indicates that up-regulation of the COX-2/PGE2 pathway induced by hypoxia would play a central role in the association of OSA and cancer.
Cannabinoid receptors [103]	Biomarkers associated with tumor progression. Specific subtypes of cannabinoid receptors (CBs) participate in several physiological processes.	CB agonists promote proliferation and aggressiveness of colon cancer cells through the activation of AKT/GSK-3β pathway. Chronic intermittent hypoxia facilitates proliferation and migration of breast cancer cells by upregulating CB1 and CB2 in vitro and in vivo.
Endostatin [104]	Potent inhibitor of tumor angiogenesis, endothelial proliferation, and tumor growth. Potential therapeutic role in cancer.	Intermittent hypoxia promotes tumor growth and cancer progression in mice models. Anti-tumor effect of endostatin in animal models under intermittent hypoxia (OSA model).
Endothelin-1 [28]	Promoter of cell proliferation and migration, angiogenesis, metastasis, and chemoresistance.	Cancer promotion role of endothelin-1 and its receptors under intermittent hypoxia conditions. In in vitro and in vivo models, endothelin receptor blockade prevents intermittent hypoxia-induced tumor development.

## 4. Genetic Factors

There is ample evidence that genetic factors influence the expression of OSA. This evidence includes reports of increased risk in first-degree relatives [106,107,108,109,110], and associations observed between genomic variants and many well-established physiological risk factors for OSA (e.g., soft tissue volumes [106], craniofacial dimensions [111,112,113,114], and obesity [115,116]). In recent years, well-powered genome-wide studies in individuals from diverse ancestral backgrounds have begun to identify variants associated with the expression of OSA, as well as objectively-measured markers of OSA severity (e.g., AHI, SpO_2_ < 90%) [117,118,119,120]. More specifically, the biological functions of genes implicated in the risk for OSA and OSA severity point to molecular mechanisms that also influence the risk of various types of cancer. This suggests that pleiotropic genetic effects underlie the relationship between OSA and several forms of cancer. Identifying and characterizing the pleiotropic effects of mechanisms overlapping between OSA and cancer could help pinpoint the underlying genetics connecting these conditions. This could be particularly useful with respect to more effective treatment options, by increasing our knowledge of how convergent mechanisms influence the risk of OSA and cancer in the same individual, and by helping to identify novel diagnostic markers and treatment targets.

One of the most consistently reported genetic findings connecting OSA and cancer is variability in the *HIF1A* gene, which encodes the alpha subunit of transcription factor HIF-1. *HIF1A* expression is mediated by hypoxia, and studies have found not only that this gene is overexpressed in many human cancers, but also that increased expression may relate to treatment failure and increased mortality in cancer patients [121]. Not surprisingly, chronic IH (CIH), which is a common physiological state in OSA, has been found to induce *HIF1A* expression—including evidence from lung cancer cell lines [122] and pre-cancerous cell models of colorectal cancer [123]. As *HIF1A* encodes for a transcription factor with a large number of susceptible downstream genes, CIH-related induction of *HIF1A* can result in the increased expression of several other genes encoding proteins involved in cancer-related pathways [122,124]. This includes activating the expression of genes known to induce malignancy (*ATAD2* (ATPase family AAA domain containing 2) [122]) and genes that have been implicated in reduced survival in lung adenocarcinoma patients (*GBE1* (glycogen branching enzyme), *HK2* (hexokinase 2) [125]). Whole-genome interrogations in humans have also begun to identify significant associations between objective respiratory measures from PSG and variants in genes that modulate *HIF1A*-mediated hypoxic-response pathways [117]. Specifically, numerous variants located in a region of the genome where the neuregulin 1 (*NRG1*) gene is encoded have proved to be associated with AHI at the level of genome-wide significance [117]. The NRG1 protein product relates to *HIF1A* as it promotes the accumulation of the HIF1A protein [126]. Furthermore, there are various tumor types that have been identified as being *NRG1*-positive, suggesting that *HIF1A* and *NRG1* have pleiotropic effects on OSA and cancer [127].

Several other OSA candidate genes, with potentially pleiotropic effects encode proteins involved in biological mechanisms that provide useful information on the relationship between OSA and cancer. These include many genes involved in glucose metabolism. For example, one recent genome-wide association study (GWAS) conducted in >500,000 individuals observed associations between variants near the lysine demethylase 2 (*KDM2B*) gene and OSA diagnosis [118]. As regards the involvement of this gene in glucose metabolism, the increased expression of the KDM2B protein has been shown to relate to increased fasting glucose [128]. Strikingly, *KDM2B* is also HIF1A-dependent, as are many lysine demethylases, and it may be upregulated during sustained hypoxia [129]. Genes encoding lysine demethylases may also play an important role in cancer, as some have been observed to be overexpressed in various types of cancers and to participate in cellular processes that are dysregulated in cancer (e.g., cell senescence, cell differentiation) [130]. Other genes that influence glucose metabolism and were implicated in the risk of OSA via this large GWAS include the brain-derived neurotrophic factor (BDNF) [118], which is known to modulate both insulin sensitivity and glucose transport [131]. It should be noted that numerous studies have investigated a potential relationship between BDNF protein levels and OSA, with varying results; in addition, a recent systematic review and meta-analysis of five studies evaluating BDNF levels in OSA compared to controls did not observe any significant difference [132]. Regardless, the BDNF protein has been reported to be upregulated in a wide range of tumors, and the pathway in which it functions has been proposed as a potential target for cancer treatment [133].

In addition to protein-coding genes, many studies have reported that microRNAs—which help regulate protein-coding gene expression—may be involved in the relationship between OSA and cancer. Several recent studies have examined differential exosomal, or circulating, microRNA (miRNA) expression in individuals with OSA [134,135,136,137]. One such study observed that miRNAs that influence cell proliferation in certain types of cancer (miR-1254 and miR-320e) demonstrated gradual increases in expression, corresponding to OSA severity as defined by PSG [134]. Another study, conducted in mouse models of lung cancer, detected several differentially expressed miRNAs that were upregulated (miR-767, miR-124-3p, miR-590-3p) and downregulated (miR-466f-5p and miR-5122) in mice exposed to IH [136]. Further evidence has suggested that IH-induced downregulation of certain miRNAs may promote tumorigenesis [135]. Some studies have also suggested that miRNAs may be involved in altering the properties of cancer cells in individuals with OSA, and, more specifically, that particular miRNAs underlie the differential responses observed in melanoma cells with serine/threonine kinase 11(*STK11*) gene mutations when exposed to exosomes from untreated OSA patients [137]. One recent study also evaluated the usefulness of the genetic testing of miRNAs for OSA screening and diagnosis [136]. Interestingly, this study performed high-throughput miRNA sequencing in patients with moderate-to-severe OSA, as well as in controls; and then, evaluated whether a diagnostic panel derived from the miRNAs with the greatest differences in expression could accurately predict OSA. A miRNA panel that was highly predictive of OSA [137] was identified, suggesting miRNA panels may be useful OSA diagnostic biomarkers.

**Figure 1:**
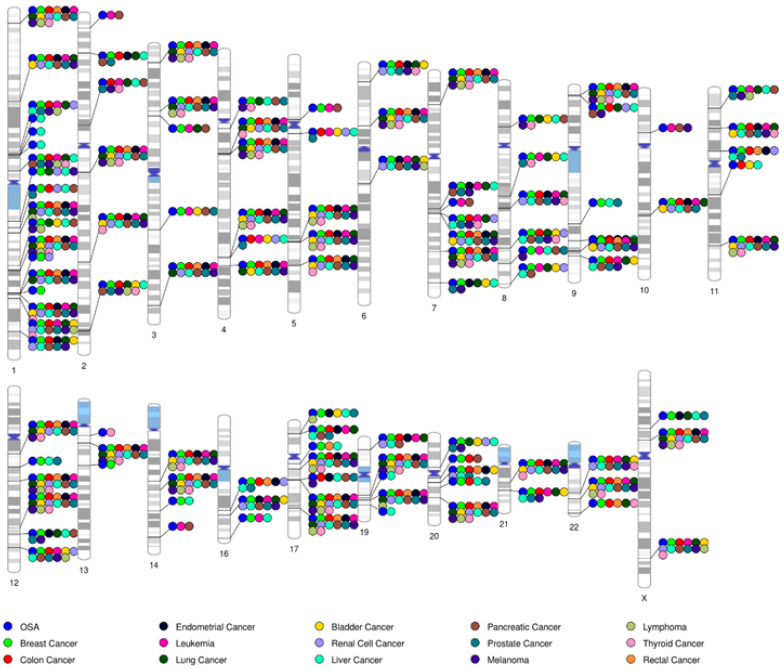
**Genes jointly implicated in OSA and prevalent cancers.** Shown are idiograms mapping genes that have been implicated in OSA to human chromosomal locations. The overlap with genes implicated in the most prevalent cancer types, based on reports from the United States National Cancer Institute, is indicated with color-coded circles. The list of OSA and cancer candidate genes were identified by querying data available in the DisGeNET7.0 database (http://www.disgenet.org accessed on 4 February 2023) and selecting associations with OSA that were reported in ≥2 distinct citations. This figure was generated using PhenoGram software (https://visualization.ritchielab.org/phenograms/plot0 accessed on 4 February 2023).

While specific genetic findings have been highlighted throughout, there are many more genetic factors with reported pleiotropic effects that connect OSA with numerous types of cancer (Figure 1). It is notable that many genes implicated in OSA—particularly those being identified using genome-wide studies aimed at the discovery of novel risk variants in humans—are also suspected of playing a role in cancer [117,118,119,120]. Additional interrogations into the genetic risk factors influencing the expression of OSA, including large-scale genetic studies, as well as further research into the involvement of epigenetic mechanisms, should continue to inform our understanding of the genetic mechanisms underlying OSA–cancer relationships.

## 5. Exosomes

Tumor cells and neighboring cells within the tumor matrix continuously communicate through a number of processes, including paracrine and autocrine processes, as well as through unique elements mediating cell–cell interactions [138,139,140,141,142,143,144]. More recently, exosomes have been suggested as one of the key mechanisms underlying the intercellular communication processes that govern alterations in the tumor microenvironment and also directly affect the biological properties of cancer cells [145,146,147,148,149]. The release of membrane-bound vesicles such as exosomes from cells is a highly conserved biological event that is ubiquitous in virtually all cells. Recent studies have discovered that transformed-tumor cells can take advantage of these endogenous ‘trafficking systems’ by transferring molecules (e.g., miRNAs) that activate cancer-related pathways; i.e., anti-apoptotic, proliferative, metabolic pathways [150,151,152,153]. Cancer cells recruit exosomes to broaden communication within the local tumor microenvironment and beyond. The processes underlying the biogenesis, release, and uptake of exosomes are tightly regulated and governed by diverse signaling mechanisms, which can be altered in pathologies such as cancer to create dysfunctional pathways. Generally, exosome biogenesis, material cargo sorting, and release involve the endosomal sorting complex required for transport (ESCRT complex), acting in conjunction with associated proteins. Plasma-derived exosomes can interact at the vascular interface of tumors and orchestrate the enrolment of endothelial, matrix, or inflammatory cells, thereby promoting the development of a tumor microenvironment with invasive and metastatic potential. MiRNAs are short, single-stranded (19–25 nucleotides in length), nonprotein-coding RNA transcripts that are initially produced in the nucleus and then, transported into the cytoplasm, where they undergo a series of steps to reach maturation. Mature miRNAs regulate gene expression by binding to the sequence of a target mRNA. This interaction results in translational repression and/or mRNA cleavage, which consequently decreases the levels of the mRNA coding protein. MiRNAs have been found to be aberrantly expressed in many diseases, including cancer; in addition, they are, as mentioned, a constitutive element of exosomes. We have recently demonstrated that mice exposed to a model of OSA increase the shedding of circulating cell-free DNA (cirDNA) into circulation, which carries epigenetic modifications that may characterize cell populations within the tumor and underlie the increase in aggressive tumor behavior [154]. Furthermore, recent studies have shown that non-coding RNAs (ncRNAs), which include long non-coding RNAs (lncRNAs) and microRNAs (miRNAs), are specifically sorted into exosomes in a very tightly regulated fashion when tumors experience hypoxia; in addition, that such ncRNAs function as important modulators of specific aspects of cancer biology, namely cell proliferation, migration, invasion, angiogenesis, immune tolerance, and drug resistance, thereby reinforcing the emerging concept that ncRNA networks synergistically respond to hypoxia to modify the properties of a cancer [155].

In light of their ubiquitous presence in bodily fluids, as well as their ability to reliably deliver genetic messages between cells and ultimately alter the phenotype of the target cells, exosomes have emerged as potentially important biomarkers of end-organ morbidity in OSA [156,157,158,159,160]; and also serve uniquely important functions in the context of solid tumors that may mediate the specifically aggravated features of cancer in patients suffering from OSA [161,162,163,164,165]. In previous studies, we showed that exosomes derived from the plasma of mice exposed to IH or fragmented sleep mimicking the hallmark perturbations of OSA displayed altered miRNA cargos, and bioinformatic analyses of their potential downstream effects further suggested that multiple cancer-related pathways would be potentially affected [166,167]. Indeed, exosomes isolated from plasma samples of patients with OSA or with obesity hypoventilation syndrome, the most severe phenotype of OSA, led to altered tumor proliferation, migration, and the invasion of various tumor cell cultures [167,168]. It is particularly interesting that two cell lines’ cutaneous melanoma were found to exhibit not only differential responses to in vitro exposures to IH, but also that exosomes released by such cell lines displayed major differences in their miRNA cargo [139]. Indeed, 46 miRNAs were differentially expressed in exosomes from CRL-1424 melanoma cells (i.e., hypoxia-sensitive cells) when compared to normoxic conditions, while only 8 miRNAs were differentially expressed in exosome CRL-1675 melanoma cells after exposure to IH. Downstream gene target pathways for these differentially expressed miRNAs further revealed cancer-related and metabolism-related pathways, such as Ras, MAPK, ErbB, AMPK, and cAMP pathways [139]. The divergent response of the cancer cells of specific organs to intermittent or sustained hypoxia should not come as a surprise and may account for some of the heterogeneity of findings in epidemiologic studies in which, for example, all lung cancers are clustered in a single, homogenous group rather than being examined separately [25].

There is no doubt that the observations linking OSA to cancer via mediator effects of exosomes are still in their very early stages; however, the prospects of identifying unique singular pathways that have relevance to the dyad of OSA and cancer are likely to be enhanced by such efforts. Furthermore, exosomes have recently begun to emerge as unique biomarkers in the context of liquid biopsy approaches to cancer, as well as providing exciting prospects of therapeutic vehicles specifically targeting tumor cells and the factors that enable their adaptive resistance strategies [169,170,171].

## 6. Microbiota

In the last decade, there has been a growing interest in the potential impact on health and disease of microbial populations inhabiting the gastrointestinal tract. It has become apparent from the extensive research literature published over the last few years that microbiota play inordinately critical roles in modulating physiological processes that include, among many functions, immune system development and responses to external challenges, pathogen displacement, and nutrient uptake, as well as brain–gut axis homeostasis, and related neurological and psychiatric functional homeostasis, including the regulation of sleep structure and function [172,173,174,175,176,177,178,179,180]. Consequently, any alterations in the composition and diversity of the microbiome (i.e., dysbiosis) have been implicated in a wide array of diseases, including cancer [181,182,183,184,185]. In the context of interactive and bidirectional relationships, colonization with specific bacterial species or diffuse changes in abundance or diversity have been correlated with either a reduced or an elevated risk of particular cancers. As such, microbiome alterations may play a direct or indirect role in oncogenesis and cancer progression by promoting chronic inflammation and producing an array of toxins and metabolites. Similarly, changes in the microbiome may disrupt the therapeutic efficacy and increase the frequency or severity of side effects from both immunotherapy and chemotherapy [164,186,187,188,189,190,191,192].

In parallel with such advances in our understanding of the microbiome and cancer, similar associations have emerged to link changes in the gut microbiome induced by the presence of OSA with end-organ morbidities of the disease [193,194,195,196,197,198,199]. Moreover, IH and sleep fragmentation impose substantial changes on the gut microbial ecosystems and elicit downstream effects that ultimately recapitulate the morbidities of OSA with great fidelity [200,201,202,203,204,205,206,207,208,209,210,211,212]. The uniquely important contributions of the changes in the microbiome to the clinical phenotypic expression of OSA have prompted a recent proposal that alterations leading to a “healthier” gut microbiome would enhance the reversal of some morbidities. Although this field is only now starting to evolve, the preliminary findings, based on the administration of pre- and pro-biotics as adjuvant treatment for OSA, are encouraging and ameliorate the specific functional deficits incurred by the presence of OSA [213,214,215,216,217]. Considering the dynamic triangular interest revolving around the microbiome, cancer, and OSA, it is only natural that the initial studies will explore the potential contributions of changes in OSA affecting the microbiome in the pathophysiology of specific cancers [124,218]. Conversely, the potential effects of sleep perturbations or cancer therapy on gut microbiota that affect cancer patients’ response to treatment are also likely to be investigated more extensively in the near future [215,219].

## 7. Conclusions and Future Challenges

The relationship between OSA and cancer has been shown to be very heterogeneous in the various clinical studies carried out to date. A greater number of pathophysiological pathways have been uncovered over time, increasing the biological plausibility of a true association between cancer and OSA, while also explaining the enormous heterogeneity of this association. A tumor’s histological type and microenvironment may be crucial to any explanation as to why the presence of OSA is only associated with a worse prognosis in some tumors and not in others—depending on the different pathophysiological pathways that are activated in response to the genetic or acquired susceptibility of different cell lines to the consequences of OSA. This response could also be modified by other factors, such as age, sex, comorbidities, and treatments.

It seems evident that, although the topic of the relationship between OSA and cancer is scientifically very attractive, many questions remain to be resolved. These include: What are the tumor types that are most affected by OSA and its consequences? What pathophysiological pathways act to a greater extent in each type of tumor in OSA patients? What role can CPAP treatment or other alternative treatments play in improving tumor progression or in reducing its incidence? What type of individuals with cancer, or a high risk of suffering from it, would require a sleep study to rule out the presence of OSA? What is the best OSA marker for an association with cancer? These and many more questions remain unanswered and must be addressed in the coming years before we can affirm that OSA is a “true” risk factor for some types of tumors, and that the treatment of OSA (with the elimination of sleep-disordered breathing and associated intermittent hypoxemia and sleep fragmentation) could impact the natural history of cancer.

## Figures and Tables

**Table 1:** Known pathophysiological links between obstructive sleep apnea and cancer.

Cell Dysfunction
Macrophage polarization
Natural killer T-cell deficiency
CD8 + T cells lymphocyte dysfunction
CD3 + gd-T-cell dysfunction
Stem-cell-like properties
Peripheral dendritic cell depletion
**Biomarkers**
VEGF and other pro-angiogenic molecules
TGF-beta 1
TNF-alpha
Tryptophan metabolism
Cyclooxigenase-2 pathway
Cannabinoid receptors
Soluble PD-L1
Endostatin
Endothelin-1 (and receptors)
Oxidative stress molecules
Paraspeckle protein-1 upregulation
**Genetic**
Glucose metabolism genes
HIF-1 gene expression (and variants)
Common key genes in OSA and cancer
Micro-RNA-320b and others
NF-kB factor genes
**Other links**
Exosomes
Microbiota

## Data Availability

The data presented in this study are available in this article.
